# Supplementary material for: Detection and Characterization of Zoonotic Pathogens in Game Meat Hunted in Northwestern Italy
Source: Animals (Basel). 2024 Feb 7;14(4):562. doi: 10.3390/ani14040562 (PMC10886018; doi:10.3390/ani14040562)
Supplement: Supplementary file 1 [file animals-14-00562-s001.zip › animals-2801715-supplementary.pdf]

SUPPLEMENTARY MATERIALS

Table S1. Genetic serogroup identification of L. monocytogenes.

**Target**	**Serotype**				
	IIa	IIb	IIc	IVb	IVa
prfa	+	+	+	+	+
prs	+	+	+	+	+
lmo 0737	+	-	+	-	-
lmo 1118	-	-	+	-	-
orf 2819	-	+	-	+	-
orf 2110	-	-	-	+	-
